# Outcomes of resections for pancreatic adenocarcinoma with suspected venous involvement: a single center experience

**DOI:** 10.1186/s12893-015-0086-1

**Published:** 2015-08-22

**Authors:** Christoph W. Michalski, Bo Kong, Carsten Jäger, Silke Kloe, Barbara Beier, Rickmer Braren, Irene Esposito, Mert Erkan, Helmut Friess, Jorg Kleeff

**Affiliations:** Department of Surgery, Technische Universität München, Ismaningerstrasse 22, 81675 Munich, Germany; Institute of Radiology, Technische Universität München, Munich, Germany; Institute of Pathology, Technische Universität München, Munich, Germany; Current address: Department of Surgery, University of Heidelberg, Heidelberg, Germany; Current address: Institute of Pathology, Heinrich Heine University Düsseldorf, Düsseldorf, Germany; Current address: Department of Surgery, Koc University School of Medicine, Istanbul, Turkey

**Keywords:** Pancreatic cancer, Venous invasion, Upfront surgery, Prognosis

## Abstract

**Background:**

Pancreatic ductal adenocarcinoma (PDAC) patients frequently present with borderline resectable disease, which can be due to invasion of the portal/superior mesenteric vein (PV/SMV). Here, we analyzed this group of patients, with emphasis on short and long-term outcomes.

**Methods:**

156 patients who underwent a resection for PDAC were included in the analysis and sub-stratified into a cohort of patients with PV/SMV resection (*n* = 54) versus those with standard surgeries (*n* = 102).

**Results:**

While venous resections could be performed safely, there was a trend towards shorter median survival in the PV/SMV resection group (22.7 vs. 15.8 months, *p* = 0.157). These tumors were significantly larger (3.5 vs 4.3 cm; *p* = 0.026) and margin-positivity was more frequent (30.4 % vs 44.4 %, *p* = 0.046).

**Conclusion:**

Venous resection was associated with a higher rate of margin positivity and a trend towards shorter survival. However, compared to non-surgical treatment, resection offers the best chance for long term survival.

## Background

The extension of the resectability criteria for pancreatic adenocarcinoma (PDAC) has been intensively discussed in the last decade. It has been shown that arterial resections significantly increase mortality [1] but that resections for presumed invasion of the superior mesenteric/portal vein axis (PV/SMV, “venous resections”) can be carried out safely without increased morbidity/mortality. In addition, taken all patients together, venous resections are not associated with an inferior survival than standard resections [2]. However, some groups have argued that true (in contrast to suspected) venous infiltration was associated with significantly shorter survival [3–6] while others have not reproduced these results [7–9]. Nonetheless the data on venous resections, especially those on perioperative outcome, have changed clinical practice in that these resections have become the standard for such tumors. Despite these technical improvements and the wide-spread introduction of adjuvant (and neoadjuvant) chemotherapy, prognosis has not much changed [10, 11].

At the same time, outcome prediction remains challenging because a considerable subgroup of patients probably does not benefit from surgery. This is because it is difficult to predict pre-/intraoperative undetectable (“subclinical”) metastatic disease and in line with this it is currently impossible to predict the biological behaviour of the tumors. Thus, there is currently a gap between our knowledge of the biology of PDAC and the technical/surgical advances. This is also underscored by a recent hypothesis that tumor size (and thus likelihood of vascular infiltration) and the time of metastasis are closely linked. Here, an exponential probability for metastatic spread was calculated depending on the size of the primary tumor [12]. Thus, many tumors deemed resectable will probably already have metastasized, though clinically not detectable [12]. In this study, we analysed our cohort of resected PDAC patients with special emphasis on venous resection/involvement and tumor biology/outcome.

## Methods

The Institutional Review Board (Ethikkommission der Medizinischen Fakultät der Technischen Universität München, Munich, Germany) approved prospective and retrospective data collection as well as tissue collection (1926/07 and 5893/13). Written informed consent was obtained from all patients. Analysis was conducted on an anonymized data set.

### Database and recorded parameters

In July 2007, we established a prospective database for the assessment of patients who underwent surgery for pancreatic diseases. The following parameters were recorded: tumor entity, age, gender, pre-operative weight loss, the following preoperative blood parameters: GPT/ALAT, GOT/ASAT, bilirubin, AP, gamma-GT and CA19-9, comorbidities (pre-operative presence of diabetes mellitus, previous or concomitant cancer diagnoses other than PDAC, a history of (acute/chronic) pancreatitis, medical co-morbidities as reflected by the ASA score), presence of preoperative jaundice and/or ERCP/bile duct/pancreatic duct stenting, treatment (e.g. surgical technique, (neo-)adjuvant therapy), intraoperative blood transfusion(s), duration of operation, resection (and reconstruction) of the portal/superior mesenteric vein, histologically confirmed presence of venous invasion, size of the tumor (T), lymph node status (N), resection margin (R), grading (G), UICC-classification, postoperative complications (according to the Clavien-Dindo classification [13]). The characteristics of the patients in the different cohorts are depicted in Table 1. Resectability criteria were defined according to the NCCN [14]. Locally advanced unresectable tumors were treated with neoadjuvant treatment protocols as decided in a multidisciplinary tumor board. Occasionally, patients were referred to our hospital after neoadjuvant or palliative intended therapy for borderline or locally advanced tumors. Lymph node dissection was standardized for all cases as described in the recent ISGP definition [15]. Venous resection was not considered a contraindication for surgery. The described approach did not change during the study period.

**Table 1:** Patients characteristics, venous resection vs. no venous resection

	Venous resection (VR) (*n* = 54)		No venous resection (NVR) (*n* = 102)		*p*-value^1^
Sex					0.24
M	27	(50.0 %)	61	(59.8 %)	
F	27	(50.0 %)	41	(40.2 %)	
Age, mean (STD). yrs	67.8	(10.7)	67.2	(11)	0.95
Preoperative performance status					
ASA					0.86
1	3	(5.6 %)	8	(7.8 %)	
2	31	(57.4 %)	58	(56.9 %)	
3	20	(37.0 %)	36	(35.3 %)	
Diabetes mellitus	16	(29.6 %)	27	(26.5 %)	0.67
Bile duct stent	18	(33.3 %)	39	(38.2 %)	0.54
Pancreatitis	2	(3.7 %)	12	(11.8 %)	0.09
Neoadjuvant treatment	4	(7.4 %)	6	(5.9 %)	0.43
Type of Surgery					0.001
pp-Whipple	25	(46.3 %)	63	(61.7 %)	
cl-Whipple	8	(14.8 %)	6	(5.9 %)	
TP	16	(29.6 %)	9	(8.8 %)	
DP	5	(9.3 %)	22	(21.7 %)	
DPH	0		2	(1.9 %)	
Vein resection technique					
End-to-End-Anastomosis	33	(61.1 %)			
Wedge-Resection	15	(27.8 %)			
goretex™ graft	6	(11.1 %)			
Operative time, mean (STD), min.	378	(103)	317	(85)	<0.001^2^
Blood transfusion	9	(16,7 %)	4	(4,0 %)	0.012
Adjuvant chemotherapy	46	(85.2 %)	87	(85.3 %)	0.97
Pathological findings					
Tumor grade of differentiation					0.28
G1	1	(1.8 %)	8	(7.8 %)	
G2	25	(46.3 %)	41	(40.2 %)	
G3	28	(51.9 %)	53	(52.0 %)	
T-stage					0.36
T1	1	(1.8 %)	4	(3.9 %)	
T2	3	(5.6 %)	7	(6.9 %)	
T3	41	(75.9 %)	83	(81.4 %)	
T4	9	(16.7 %)	8	(7.8 %)	
Tumor size in cm (median, min-max)	4.3	1.5-12	3.5	1.2-8.5	0.026^3^
Nodal status					0.82
0	16	(29.6 %)	32	(31.4 %)	
1	38	(70.4 %)	70	(68.6 %)	
Number of lymph nodes (median, min-max)	21	(5–62)	20	(7–54)	0.92
Resection margins					0.046^4^
R0	22	(40.8 %)	60	(58.8 %)	
R1	24	(44.4 %)	31	(30.4 %)	
R2	0		1	(1.0 %)	
Rx	8	(14.8 %)	10	(9.8 %)	
Pathology report: venous infiltration					
no	8	(14.8 %)			
yes	19	(35.2 %)			
unkown	27	(50.0 %)			

^1^ *X* ^2^-test; ^2^Student’s *t*-test; ^3^Mann–Whitney-*U*-test; ^4^ *X* ^2^-test: R0 vs. R1

Abbreviations: *pp-Whipple* pylorus-preserving Whipple (=pp-partial pancreaticoduodenectomy), *cl-Whipple* “classical” Whipple, *TP* total pancreatectomy, *DP* distal pancreatectomy, *DPH* DP plus partial pancreatic head resection, *STD* standard deviation

### Inclusion and exclusion criteria

**Fig. 1:**
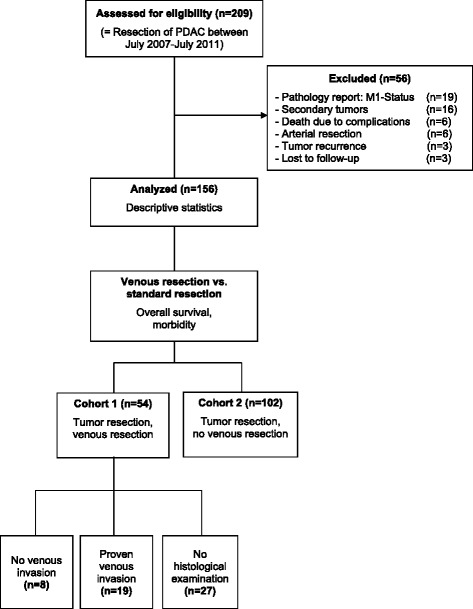
Inclusion and exclusion criteria. Flow-chart of inclusion and exclusion criteria. The prospective database was retrospectively searched according to the criteria as described in the Methods section. Two cohorts of patients were defined: short- and long-term survivors and patients on whom standard resections or resections with vein resections were performed

We retrospectively identified 209 patients who underwent an elective pancreatic resection with a final histopathological diagnosis of PDAC between 07/2007 and 07/2011 (Fig. 1). The following patients were excluded from the analysis: UICC stage IV disease (*n* = 19), patients who were resected for local tumor recurrence (*n* = 3), patients on whom arterial resections were performed (*n* = 6), patients with a history of another cancer disease (*n* = 17) and patients who died because of surgery-related complications within 4 months from surgery (extension as suggested by Strasberg et al. [16], *n* = 6); three patients were lost to follow-up. Due to the relatively small patient cohort and considering the 2:1 ratio of the non-vein resected to the vein resected group, we opted for the inclusion of all patients and against a matched case analysis.

At the time of analysis, 17 out of 54 patients in the group of vein-resected patients and 34 out of 102 patients in the standard resection group were alive. Median follow-up for all patients was 20.8 months (3.3-69.8 months) and median follow-up for patients alive was 37.7 months (21.0-69.6 months). Patient charts were reviewed for whether or not a resection of the PV/SMV (wedge resection, complete resection with end-to-end anastomosis or interposition of a goretex™ graft) had been performed. In this cohort of patients, preoperative imaging (computed tomography of the abdomen or magnetic resonance imaging/magnetic resonance cholangiopancreaticography (MRI/MRCP)) was evaluated to estimate sensitivity and specificity in regard to defining the presence of true venous invasion (versus an inflammatory/desmoplastic reaction at the PV/SMV).

### Statistics

Continuous variables are reported as means ± SD or median (min.-max./95 %-CI), and were compared using a Student’s *t*-test or a Mann–Whitney-*U*-test, as appropriate. Categorical variables are summarized as frequency counts and percentages and were compared using Fischer’s exact test or Pearsons’s chi-square test, as appropriate. Overall survival was defined as time from resection until death or until last follow-up.

Survival analysis was performed using the Kaplan-Meier method; differences were evaluated with the log rank test. A two-sided P value of <0.05 was considered as significant. All statistical analyses were performed using IBM SPSS, v20 for Windows (IBM Inc., USA).

## Results

### Analysis of venous resection

The cohort of patients on whom venous resections have been performed was compared with the cohort of patients on whom standard pancreatic resections have been performed (Table 1). Except for late portal vein thrombosis (7 cases in the venous resection cohort versus one case in standard resection group), postoperative complications and (early and late) mortality were comparable in both groups (Table 2). These findings are in line with published data [2, 7, 17, 18], demonstrating that venous resections can be performed safely. Interestingly however, significantly more resections were margin-positive (i.e. R1; 44.4 % vs. 30.4 %, *p* = 0.046) in the venous resection cohort of patients. This might be a reflection of larger tumors in the patients with venous resections (4.3 cm vs. 3.5 cm, *p* = 0.026, Table 1). While some groups demonstrated that true tumor cell invasion of the PV/SMV negatively impacted on prognosis [3–6, 19], others have denied such an association [7–9]. We thus re-assessed the histo-pathology reports on invasion/no-invasion of the vein (Table 1). In those cases were data on vein invasion was available, 30 % were tumor cell-negative whereas in 70 %, tumor cell invasion of the vein was seen. We then analyzed the patients with confirmed invasion of the vein vs. no-invasion; here, no significant differences in regard to preoperative markers, operative time or surgical technique were found (Table 3). Comparable to the whole cohort of venous resection patients, postoperative complications (including late surgical related mortality) occurred at similar frequencies in both groups. Further analysis however demonstrated that the tumors in the true venous invasion group of patients were significantly more advanced (T3/4 tumors in the no-venous invasion vs. venous invasion groups: 4/8 vs. 19/19, *p* = 0.001, Table 3). In line with these findings that implicated more advanced tumors in the true invasion group, significantly more patients had lymph node metastases when the vein was truly infiltrated (Table 3). CT or MRI scans were available in our digital imaging system from 23 of 27 patients. Radiological assessment of these scans revealed that CT or MRI predicted true invasion of the vein only at a sensitivity of 69 % and a specificity of 29 % (Table 3). Though the patient number is low, these data demonstrate the difficulties in preoperative prediction of the presence/extent of PV/SMV invasion.

**Table 2:** Postoperative complications (30-days), venous resection vs. no venous resection

		Venous resection VR (*n* = 54)		No venous resection NVR (*n* = 102)	*p*-value^1^
Postoperative complications^2^					0.38
Grade I	12	22.2 %	15	14.71 %	0.19
Grade II^3^	19	35.2 %	38	37.3 %	0.86
Grade III	8	14.8 %	9	8.8 %	0.28
Grade IV	1	1.8 %	1	1.0 %	1.00
120 day mortality^4^	3	5.3 %	3	2.9 %	0.37
Portal vein thrombosis					
30 days	1	1.8 %	1	1.0 %	0.69
total	7	13.0 %	1	1.0 %	0.001
Pancreatic fistula, grade C	2	3.7 %	4	3.9 %	
Lymph fistula	2	3.7 %	3	2.9 %	
Delayed gastric emptying	6	11.1 %	4	3.9 %	0.09
Diarrhea	4	7.4 %	4	3.9 %	
Intraabdominalabscess	2	3.7 %	5	4.9 %	
Cholangitis	3	5.6 %	15	14.7 %	0.11
Wound infection	3	5.6 %	8	7.8 %	
Liver ischemia	1	1.8 %	0	0.0 %	
Bleeding	2	3.7 %	0	0.0 %	
Re-operation	2	3.7 %	3	2.9 %	
Pneumonia	0	0.0 %	1	1.0 %	
Urinary tract infection	2	3.7 %	2	2.0 %	
Cardiac dysfunction	1	1.8 %	3	2.9 %	

^1^ *X* ^2^-test

^2^Clavien-Dindo-Classification

^3^VR: 9 patients (47.4 %) only blood transfusion. 6 patients (31.5 %) antibiotics. 1 patient (5.3 %) blood transfusion and antibiotics; without VR: 4 patients (10.5 %) only blood transfusion. 21 patients (55.3 %) only antibiotics; 4 patients (10.5 %) blood transfusion and antibiotics

^4^VR (*n* = 57) vs. NVR (*n* = 105)

**Table 3:** Characteristics of 27 patients with vein resection and confirmed pathology report

	No venous invasion (*n* = 8)		Venous invasion (*n* = 19)		*p*-value^1^
Sex					0.33
M	5	(62.3 %)	8	(42.1 %)	
F	3	(37.4 %)	11	(57.9 %)	
Age, mean (STD), yrs	61.7	(13.2)	65.0	(10.3)	0.48
Preoperative performance status					
ASA					0.23
1/2	4	(50.0 %)	14	(73.7 %)	
3	4	(50.0 %)	5	(26.3 %)	
Diabetes mellitus	1	(12.5 %)	6	(31.6 %)	0.41
Bile duct stent	2	(25.0 %)	11	(57.9 %)	0.23
Type of surgery					0.55
ppWhipple/cl-Whipple	6	(75.0 %)	12	(63.2 %)	
TP	2	(25.0 %)	7	(36.8 %)	
Vein resection technique					0.19
End-to-End anastomosis	6	(75.0 %)	15	(78.9 %)	
Wedge- Resection	2	(25.0 %)	1	(5.3 %)	
goretex™ graft	0		3	(15.8 %)	
Operative time, mean (STD), min.	416	(42)	379	(85)	0.222^2^
c^3^					0.52
Grade I/II	4	(50.0 %)	11	(57.9 %)	
Grade III/IV	2	(25.0 %)	4	(21.1 %)	
Pathological findings					
Tumor grade of differentiation					0.47
G1/G2	6	(75.0 %)	9	(47.4 %)	
G3	2	(25.0 %)	10	(52.6 %)	
T-stage					0.001
T1/T2	4	(50.0 %)	0		
T3/T4	4	(50.0 %)	19	(100.0 %)	
Nodal status					0.038
N0	6	(75.0 %)	6	(31.6 %)	
N1	2	(25.0 %)	13	(68.4 %)	
Resection margins					0.36
R0	4	(50.0 %)	6	(31.6 %)	
R1 (incl. RX)	4	(50.0 %)	13	(68.5 %)	
Localization of tumor					0.099
Pancreatic head	7	(87.5 %)	14	(73.7 %)	
Multifocal	1	(12.5 %)	5	(26.3 %)	
Pre-operative prediction venous infiltration					
CT/MRT+	5		11		Sensitivity: 0.69
CT/MRT-	2		5		Specificity: 0.29

^1^ *X* ^2^-test; ^2^Student’s *t*-test; ^3^Clavien-Dindo-Classification

Abbreviations: *pp-Whipple* pylorus-preserving Whipple (=pp-partial pancreaticoduodenectomy), *cl-Whipple* “classical” Whipple, *TP* total pancreatectomy, *STD* standard deviation

### Survival following venous resections

**Fig. 2:**
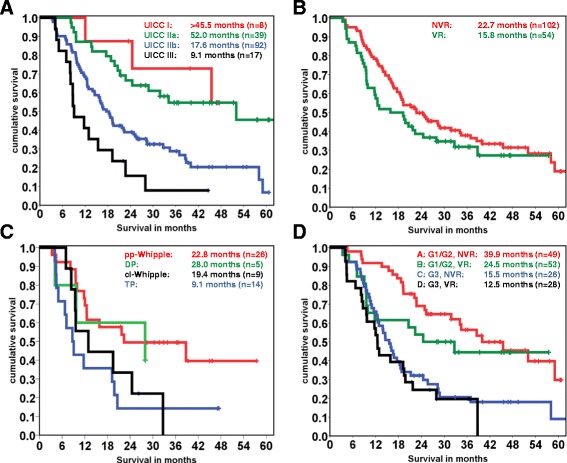
Survival analysis. **a**, Survival according to the UICC stages was assessed using the Kaplan-Meier method and the log rank test. The overall *p*-value is 0.001. **b**, Survival in the patient groups with standard (no venous resection, NVR) and with venous resection (VR) was compared using Kaplan-Meier curves and the log rank test; *p* = 0.157. **c**, Survival according to different operative techniques was assessed using Kaplan-Meier curves and the log rank test. TP: total pancreatectomy; DP: distal pancreatectomy; pp-Whipple: partial, pylorus-preserving pancreaticoduodenectomy; cl-Whipple: “classical” Whipple, partial pancreaticoduodenectomy. **d**, Comparisons between the following groups were done using the Kaplan-Meier method and log rank testing: G1/2 without vein resection (**a**), G1/2 with vein resection (**b**), G3 without vein resection (**c**), G3 vein resection (**d**). The overall *p*-value is <0.001. **a** vs **c**: *p* < 0.001; **b** vs **d**: *p* = 0.023

To determine the extent of selection bias in the venous resection cohort of patients, we stratified these according to the UICC classification and plotted the subgroups following Kaplan-Meier survival analysis (Fig. 2a). This analysis demonstrates that the general prognosis of this cohort of patients is accurately predicted by the UICC. Thus, we assumed that selection bias was not overly strong in our cohort of patients and considered that the following analyses were statistically justified.

Though there was a trend towards shorter survival of patients in whom venous resections had to be performed, this was not statistically significant (median survival 22.7 vs. 15.8 months, Fig. 2b; *p* = 0.157). Pathologically confirmed invasion of the vein was not relevant for prognosis. In addition, patients on whom a total pancreatectomy had to be performed lived shorter than those with pancreaticoduodenectomy or distal pancreatectomy (9.1 months versus 19.4 - 28 months, Fig. 2c).

Next, we analyzed tumor grade as a surrogate marker for tumor biology. Patients with well- and moderately differentiated PDACs lived significantly longer than those with poorly differentiated tumors (overall *p* < 0.001, median survival 39.9 vs. 15.5 months, *p* < 0.001; Fig. 2d), and this stratification was also significant in the venous resection cohort (median survival 24.5 vs. 12.5 months, *p* = 0.023).

## Discussion

The data presented in this paper confirm previously published results on portal vein resections during surgery for pancreatic adenocarcinoma, i.e. that it can be performed safely without increased morbidity and (early and late) mortality. There was a trend towards shorter survival and more frequent margin-positivity in the group of patients in whom venous resections had been performed. However, this was mainly a result of the outcomes after combined total pancreatectomy and portal vein resection. Such operations were only performed if a pancreaticoduodenectomy or a distal pancreatectomy were technically impossible or not sufficient to achieve macroscopic tumor clearance. This is also reflected in the finding that outcomes were worse in larger tumors. For partial pancreaticoduodenectomy and distal pancreatectomy with venous resections, there were no considerable differences compared to the respective standard resections. Thus, our data are in line with previous publications and support the concept that partial resection of the PV/SMV is justified to achieve negative margins and thereby better local control [20, 21]. However, as patients undergoing total pancreatectomy plus PV/SMV resection had worse outcomes, it might be argued that this group of patients with centrally located, large tumors, for whom total pancreatectomy is anticipated/likely, should not be operated upfront, but might be better candidates for neoadjuvant treatment.

Our study also highlights the prognostic importance of tumor biology, using grading as a surrogate marker. Obviously, tumor biology rather than tumor location (and vascular abutment/invasion) is a stronger prognostic factor. This is also supported by recent data on long term survival after PDAC resections. Here, tumor biology was identified as the most important factor, with a significant number of long term survivors having a positive margin status and lymph node metastasis [11]. Further studies are required to better predict and understand tumor biology and stratify patients that benefit most from extensive surgical procedures.

There are some drawbacks of the present study. Although standardized histopathological reporting with special emphasis on resection margins had been introduced in our center in 2007 [22–24], venous involvement had not been assessed in all cases. Thus, data regarding preoperative diagnostic accuracy and true venous infiltration have to be interpreted cautiously.

## Conclusion

In summary, our data support the current clinical practice of operating on patients with suspected involvement of the PV/SMV, and to perform (partial) resection of the PV/SMV if necessary to achieve tumor-free margins.
